# A sociogenomic perspective on neuroscience in organizational behavior

**DOI:** 10.3389/fnhum.2014.00084

**Published:** 2014-02-25

**Authors:** Seth M. Spain, P. D. Harms

**Affiliations:** ^1^School of Management, State University of New York at BinghamtonBinghamton, NY, USA; ^2^Department of Management, University of Nebraska - LincolnLincoln, NE, USA

**Keywords:** behavioral genetics, epigenetics, leadership, personality, adult development, evolutionary psychology, organizational behavior

## Abstract

We critically examine the current biological models of individual organizational behavior, with particular emphasis on the roles of genetics and the brain. We demonstrate how approaches to biology in the organizational sciences assume that biological systems are simultaneously causal and essentially static; that genotypes exert constant effects. In contrast, we present a sociogenomic approach to organizational research, which could provide a meta-theoretical framework for understanding organizational behavior. Sociogenomics is an interactionist approach that derives power from its ability to explain how genes and environment operate. The key insight is that both genes and the environment operate by modifying gene expression. This leads to a conception of genetic and environmental effects that is fundamentally dynamic, rather than the static view of classical biometric approaches. We review biometric research within organizational behavior, and contrast these interpretations with a sociogenomic view. We provide a review of gene expression mechanisms that help explain the dynamism observed in individual organizational behavior, particularly factors associated with gene expression in the brain. Finally, we discuss the ethics of genomic and neuroscientific findings for practicing managers and discuss whether it is possible to practically apply these findings in management.

It seems that we have a fascination with the brain. In *The psychopath inside*, neuroscientist James Fallon describes his discovery that scans of his own brain showed patterns of activation indicating potential psychopathy (Fallon, 2013), evocatively described as similar to scans of convicted killers. Fallon describes the neuroanatomical features associated with the constellation of behavioral tendencies that make up psychopathy, including impulsivity and lowered empathy, as well as their genetic and epigenetic correlates. This description almost immediately gives rise to questions of how determined a complex behavioral phenomenon, such as psychopathy, is by its biological foundations (see Stromberg, 2013, for a discussion of the book). Psychopathy—the tendency to be impulsive, manipulative, anti-social, and to lack fear and empathy (e.g., Hare, 1985, 1999)—is of increasing interest in the organizational sciences (e.g., Spain et al., 2014), because it can help explain phenomena such as supervisors who behave in an abusive manner toward their subordinates (cf., Krasikova et al., 2013), *managerial derailment*, the phenomenon of seemingly promising managers who become decidedly ineffective, usually due to interpersonal problems (Leslie and Van Velsor, 1996; cf., Harms et al., 2011), and *counterproductive work behaviors*, or those times when employees engage in activities such as stealing from the company, sabotage, or interpersonal aggression at work (O'Boyle et al., 2011). If organizational scientists could reliably identify psychopaths from objective indicators, such as functional magnetic resonance imaging scans of their brains or genetic tests, they may be able to design interventions that could help remediate a great deal of suffering in work organizations.

It is, however, unlikely that we can make such identifications reliably. The question posed above, how determined are complex social behaviors by their biological foundations, remains. For instance, consider the case of James Fallon above. He describes himself as a “prosocial psychopath,” and attributes his relatively benign, if competitive, behavior to growing up in a loving family (Stromberg, 2013): he has psychopathy “in his genes,” but it is not so clearly expressed in his behavior.

Additionally, there are many reasons why, even if we could make such identifications, we would not want to use this in the day-to-day practice of management. For instance, genetic screening or brain imaging could be expected to lead to a form of “genetic discrimination.” Such discrimination may be problematic for ethical reasons, and practically, as long as the biological indicators measured are weakly predictive of behavior. For instance, in the United States, the Genetic Information Nondiscrimination Act of 2008 (GINA) Title II prevents employers (and some other non-employment agencies) from requiring or requesting genetic information as a condition of employment (www.genome.gov/10002077). In spite of such legislative barriers to direct use of biological research in employment settings, interest among organizational researchers remains high, as evidenced by the forthcoming book edited by Collarelli and Arvey, *The biological foundations of organizational Behavior.*

Arvey and colleagues (Arvey et al. (2014), Arvey and Bouchard, 1994; Ilies et al., 2006) provide detailed summaries of the research on behavioral genetics in organizational behavior. The earliest investigations in behavioral genetics in organizational settings found that heritable, genetic factors accounted for variation in behavioral characteristics related to leadership (e.g., Johnson et al., 1998). More recent studies aim to examine mediators of the genetic effects, or environmental moderators of these effects to examine how inherited factors play a role in becoming a successful leader. We note that much of the organizational research using behavioral genetics has been directed at the question, “are leaders born or made,” that is, whether leadership is substantially heritable or not. Therefore, our review will be focused on leadership phenomena, but not exclusively about them.

The “are leaders born or made” question is an example of a very common question about genetics in the social sciences, which is: what wins, nature or nurture? Unfortunately, this question is effectively a straw man, because human action is the result of *both* nature and nurture (Ridley, 2003; Rutter, 2006)—one may as well ask which contributes more to the area of a rectangle, its length or its width (an analogy attributed to Donald Hebb in many sources, including Meaney, 2004, p. 2). That is, variation in almost every individual difference studied in psychology is partially due to both genetic and environmental effects. This concept has been codified in Turkheimer's three laws of behavior genetics (Turkheimer, 2000, p. 160):

1. All human behavior traits are heritable (genetic effect),
2. The effect of being raised in the same family (shared environment) is smaller than the effect of genes, and
3. A large percentage of the variation in human behavioral traits is not accounted for by either genes or by shared environment (unique environment),

which together show that genes and experience, especially an individual's *unique* experience, both play important roles in the development of complex behavioral characteristics. By this logic, leaders are both born *and* made. An additional important point is that it is meaningless to take the slightly more nuanced position of “if both are important, which is more important?” In the current essay, we review the etiology of leadership through the lens of sociogenomics. For these purposes, we consider leadership largely at the level of the individual leader—the individual characteristics and behaviors that allow that individual to emerge, be accepted, and be effective as a leader. However, each stage of this process involves social interactions with other people. We therefore do consider the influence that individuals have on one another. So, while our perspective speaks most directly to behavioral and trait-based approaches to leadership, the overarching perspective has some bearing on interpersonal and dyadic perspectives, as well.

The sociogenomic framework articulates the mechanisms through which genes and environments interact to help shape observed behavioral characteristics. The promise of sociogenomics lays in using the genome–the entirety of an organisms' hereditary characteristics–as the basis for understanding behavior (Robinson, 2004; Robinson et al., 2005; Roberts and Jackson, 2008), including leadership behavior. This paper argues that such an approach has a great deal to offer to the study of leadership, even for researchers that do not aspire to collect biological data, because the theory has broad impact on basic research questions in leadership. We believe that a sociogenomic perspective can serve as a meta-theoretical backdrop for leadership scholars that could help to integrate many disparate findings.

We contrast the sociogenomic view with three contemporary perspectives on the biological substrate of leadership: 1. The existence of genetic effects indicates that leaders are “born,” not made (e.g., Ilies et al., 2006; De Neve et al., 2012), 2. the proportionally low variance in phenotypes (about 30%) accounted for by genetic factors indicates that leaders are “made,” not born (Avolio, 2005; Avolio et al., 2009), and 3. an interactionist perspective that acknowledges the mutual influence of genetic and environmental factors (e.g., Arvey et al., 2007; Zhang et al., 2009b). Researchers tend to focus on questions driven by the first two positions. For instance, a researcher may be interested in establishing how much of the observed variance in a leadership characteristic—most of this research has focused on attaining a leadership role—is attributable to genetic factors by estimating the heritability coefficient, *h*^2^ (described below). Another researcher may be concerned with showing that some early life experience influences these same leadership characteristics. In contrast, the sociogenomic approach embraces both of these explanations simultaneously.

We also see something like sociogenomics as an effectively necessary component of doing any biological research into human social behavior in the *post-genomic* world (Charney, 2012). That is, many of the assumptions that earlier work in behavioral genetics has rested on have been called into question as a result of findings since the mapping of the human genome in the early part of this century. Most importantly, DNA is a dynamic entity whose structure and function is altered throughout the life-course by other entities such as retrotransposons (mobile DNA elements that “copy-and-paste” themselves into other sections of a person's DNA sequence; Charney, 2012) and copy number variations (deletion, insertion, and duplication mutations). Further, DNA may not be the only heritable biological element—*epigenetic* information (loosely speaking, information about how the cellular environment regulates the expression of DNA; we will describe epigenetics in more detail below) may also be transgenerationally heritable (Zhang and Meaney, 2010; Charney, 2012). Each of these elements seems to be environmentally responsive, which goes some way to explaining how the environment interacts with the genome to produce behavior.

It is important to clarify from the outset how sociogenomics differs from more traditional interactionist viewpoints; in fact, it is not the case that sociogenomics *is* an interactionist approach. It is, rather, a framework for understanding how gene × environment interactions operate; for explaining genetic and environmental effects within a common language. That is, sociogenomics *subsumes* interactionist approaches. We believe that the sociogenomic perspective provides a broader view than the basic interactionist perspective allows. Furthermore, the sociogenomic model predicts that both factors, genes and environmental experiences, work in the same way: by influencing which genes code for proteins at a given moment in time. That is, the sociogenomic approach adds value by explaining that both genes and environments operate on the genome; they both work by affecting gene expression (Robinson, 2004). In the example above, what distinguishes a sociogenomic explanation from other interactionist perspectives is the understanding that both the genetic factors and the early life experiences operate on the genome; they modulate the expression of certain genes. Sociogenomic explanations focus on *how* gene by environment interactions work.

A sociogenomic model of leadership provides an integrative framework for explaining the roles that genetics and environmental factors play in leader behavior. We next provide a brief overview of the methods used in behavioral genetics studies, called biometric models. Then we review the behavioral genetics literature on leadership, and interpret these findings in a standard behavioral genetics way. We then explain how the sociogenomic approach differs from a behavioral genetic approach. Finally, we outline a series of proposals for innovative research in leadership that are suggested by the sociogenomic model. We conclude by examining ethical considerations for practicing managers. We begin by discussing the roles of psychological and biological explanation in the study of leadership and other organizational behavior.

## Weak vs. strong biologism

Materialism is a basic tenet in much of modern philosophy, and certainly in the sciences (Dennett, 1991). That is, it should be uncontroversial to describe any human behavioral phenomenon as “biological” in the sense that our psychological selves are situated in our bodies, and therefore must run, like software does on a particular piece of hardware, on our brains—our minds live in our brains. This is the position that Turkheimer (1998) called *weak biologism*, and considered it essentially tautological, that this position is a necessary consequence of the materialist point of view. That is, since our behaviors occur through the workings of all of our bodies' biological (e.g., musculoskeletal, neurological) systems, that there is some psychobiological association is unsurprising. Where there is interest is in the position Turkheimer (1998) called *strong biologism*, that there is a strong association between well-defined biological structures or processes and well-defined human behaviors. That is, strong biologism provides the necessary mechanisms to identify the etiology of a behavioral syndrome.

The conflation of weak and strong biologism has led to much of the confusion, difficulty, and acrimony in the nature-nurture debate (Turkheimer, 1998). We believe that this is also the case in discussions of biological underpinnings in organizational behavior and leadership. For instance, in asking the question, “are leaders born or are they made?” we are implicitly asking a strong biological question—at least when the question is considered in a genetic vs. environmental causation way. That is to say, this question assumes that there is a specific biological etiology for the behavioral syndromes of leadership, a reasonably simple mechanism or set of mechanisms or processes that is localized in the brain, or there simply is not (the former position embodies the conception of leaders being born, the latter, made).

In other words, if this proposition were true, it would be possible to study leadership at the biological level of analysis, and such study would scale directly to the behavioral level. With a phenomenon as complex as leadership, this is unlikely to be the case. Such questions are not answered by examining whether a phenotypic trait is heritable (Kempthorne, 1978; Turkheimer, 1998). Estimated heritability is, however, the mainstay of our knowledge of the biological foundations of complex behavioral phenomena, including leadership and other characteristics of interest in organizational behavior. We next review the basic models of such biometric research, with the intent to make these models completely accessible to non-specialists.

## Biometric modeling

In order to understand the literature that genetic research in leadership is built on, it is necessary to understand *biometric*, or behavioral genetic, models. The standard model in behavioral genetics is defined by the equation (e.g., Plomin et al., 2001):

(1) P=A+C+E

with the components of the equation estimated using a sample of identical (*monozygotic*) and fraternal (*dizygotic*) twins. In the equation, P is the *phenotypic trait*. Phenotype means that the trait is observed or measured. Examples of phenotypic traits are height, eye color, measured intelligence, or the occupation of a leadership role. The A-term refers to the *additive* genetic component. The C-term refers to *common environment*, factors that are not genetics that make twins more alike. Typically, these factors are considered related to family upbringing and common schooling or early life experiences shared between twins. The E-term represents *unique environment* (confounded with error), or the percentage of variance attributable to experiences that are wholly unique to each individual, and other purely idiosyncratic variance. This model is typically estimated using samples of identical and fraternal twins, though adoption studies are sometimes used. Identical twins share roughly 100% of their genetic material—copy number variations can differ across identical twins, and random mutations can occur during development, but for practical purposes, identical twins share 100% of their genetic material, while fraternal twins are no more similar genetically than any other siblings—sharing on average 50% of their genetic material. Therefore identical twins have perfect genetic correlations, whereas fraternal twins have genetic correlations half as strong. Both types of twins have equally strong common environmental effects, and the unique environmental effect is specific to each individual. This model allows behavioral genetics researchers to estimate the heritability coefficient, *h*^2^, which is the population-level variance in the phenotypic trait, P, that is associated with the variance in genetic material (i.e., Kempthorne, 1978, p. 11): *h*^2^ = Var(A)/Var(P). It is extremely important to note that the *h*^2^ coefficient is a population statistic—it does not apply at the individual level, so it should never be interpreted that an *h*^2^ of 0.50 means that half of an individual's trait level is genetic. The statistic only indexes the population's proportion of phenotypic variance attributable to genotypic variance.

## Behavioral genetics in organizational behavior and leadership

Several studies have investigated the heritability of leadership styles and occupancy of leadership roles (such as supervisor or manager). For instance, Johnson et al. (1998) examined the heritability of self-reported scores on the Multidimensional Leadership Questionnaire (MLQ; Bass and Avolio, 1991). They found a heritability coefficient of 49% for transactional leadership style, and a heritability coefficient of 59% for transformational style. Again, these findings do not mean that half of any one person's score on transformational or transactional leadership is attributable to their specific genetic makeup. These findings also do not imply heritability such that leadership is, “like father, like son,” as heritability estimates do not ensure large correlations across generations (Jackson et al., 2011). More importantly, these findings *absolutely do not* suggest that leadership cannot be taught, as heritability does not imply immutability. Instead, these findings only imply that identical twins are more similar on the transformational and transactional leadership scales than fraternal twins due to inherited genetic factors.

Extending these findings within the same sample of twins, Johnson et al. (2001) examined the genetic correlations between transformational and transactional leadership styles, again measured with the MLQ, and the five factor model of personality (Goldberg, 1993). Such a design allows the researcher to determine how much of the correlation between two measured variables is determined by shared genetic causes. For example, we might estimate how much of the observed relationship between the personality trait extraversion and transformational leadership is a result of these two characteristics sharing common genetic causes. These researchers found substantial genetic correlations between transactional leadership and Conscientiousness, Extraversion, and Agreeableness (−0.49, −0.46, and −0.23). Similarly, transformational leadership was strongly genetically correlated with Conscientiousness, Extraversion, and Openness to Experience (0.58, 0.23, and 0.56). The pattern, but not the strength, of relationships was the same for both phenotypic and genotypic correlations.

Again, these correlations are at the genetic level, so it is likely that transactional leadership shares some of its underlying genetic substrate with Conscientiousness, Extraversion, and Agreeableness, while transformational leadership shares genetic substrates with Conscientiousness, Extraversion, and Openness. Specifically, based on this study, transformational leadership appears to have genetic causes in common with Conscientiousness, Extraversion, and Openness. One possible avenue for future research that these findings suggest is that any physiological system that is implicated in one of these personality traits may be a candidate for study with leadership style. For instance, the serotonin system has been implicated in self-control or impulsivity (Carver et al., 2008), so it appears relevant to conscientiousness. Therefore, it is a reasonable neurological system to study in relation to rated transformational and transactional leadership.

In a study of leadership emergence, as defined by the attainment of leadership roles such as supervisor or manager, Ilies et al. (2004) meta-analytically estimated the percentage of variance in leader emergence attributable to genetic factors, as mediated by personality traits. The results of this meta-analysis provided 17% as a lower-bound estimate of the heritability of leader emergence. This meta-analysis also provided evidence that personality traits mediate the influence of genes on leader emergence, such that genes → personality → leader emergence, as causal structure consistent with the “leaders are born” thesis (see Figure 2 and related discussion below).

In a twin study, Arvey et al. (2006) found that 30% of the variance in leadership role occupancy was explained by genetic factors, with the rest explained by non-shared environmental factors. Additionally, genetic factors accounted for substantial amounts of variance in personality traits, though there was no evidence that these personality traits mediated the genetic influence on leader role occupancy. In other words, both personality traits and leader role occupancy had heritable components, but there was no evidence in this study that the genetic effect on leadership was mediated by personality.

Additional evidence was provided by Arvey et al. (2007), who found that 32% of the variance in leader role occupancy was attributable to genetic factors. This study also tested whether developmental factors, specifically formal work experience and family experience, accounted for variance in leader role occupancy. These experiential variables both had significant zero-order correlations with leader role occupancy, but when the genetic factors were controlled for, only the work experiences factor remained associated with leader role occupancy. In other words, family experiences no longer count when genetics are controlled for, but on-the-job work experiences still independently contribute to leader role attainment.

None of these studies found that leadership, however defined, is entirely explained by genetic factors, leaving a lot of room for environmental factors as explanations. Still, leadership, however defined, has been found to have substantial genetic component with around 30–60% of the variance explained by genetic factors. The fact that a sizable amount of variance is explained by genetic factors is consistent with a “Leaders are born” approach. On the other hand, around 40–60% of the variance in self-reported leadership style and 70% of the variance in leader role occupancy was not explained by genetic factors, consistent with a “leaders are made” explanation. That work experiences contributed, independently of genetic factors, to attaining a leadership role (explaining 17% of the variance in leader role occupancy; Arvey et al., 2007), offers support for the “made” interpretation.

Additionally, Avolio et al. (2009) reported findings that after controlling for genetic effects, there were still significant effects on leader role occupancy for authoritative parenting and rule-breaking behaviors in childhood. Further, Ilies et al. (2006) reported the results of an unpublished study by Arvey et al. (2004) that found experiencing leadership roles in high school moderated the genetic effect on work leadership. These findings raise the possibility that the heritability of work leadership may be affected by environmental variables, in this particular case, earlier investment in leadership roles (Avolio, 1994). In addition, Zhang et al. (2009b) found in a sample of male twins that growing up in an enriched environment (as indicated by family socioeconomic status, perceived parental support, and reported conflict with parents) significantly moderated the heritability of leader role occupancy. Specifically, higher levels of enrichment were associated with lower heritability estimates.

The previous finding is very similar to the evidence that the heritability of cognitive ability is moderated by socioeconomic status (Turkheimer et al., 2003). Specifically, at low levels of SES, 60% of the variance in measured cognitive ability is attributable to shared environment, with almost no genetic component. At high levels of SES, the results are almost exactly the reverse, with the genetic component taking over. Taking these findings together appears to show that the enrichment of the environment that a person grows up in is an important moderator of genetic effects on a wide range of variables, including leadership style.

This latter set of findings demonstrates that, while there is a genetic component to leadership, the environment clearly has a role to play. So, the simple question of whether leaders are born or made has a very simple answer: Yes, leaders are both born *and* made. The question now shifts; was Avolio (2005) correct in emphasizing *made* over *born* in leadership development? We address this more nuanced question by examining traditional biological models of traits with a sociogenomic approach, and considering the implications of each viewpoint on the evidence thus far.

## A sociogenomic perspective

Recent advances in biology show that the “born, not made” viewpoint cannot be entirely correct (e.g., Robinson, 2004; Robinson et al., 2005; Roberts and Jackson, 2008), for any behavioral domain. That is, “When it comes to behavior, we have moved beyond genetic determinism. Our genes do not lock us into certain ways of acting; rather, genetic influences complicated and mutable and are only one of many factors affecting behavior,” (Jasny et al., 2008). The perspective we call *sociogenomic* rests on two main findings and one fundamental assumption. The assumption is taken from a sociobiological perspective of evolution (e.g., Wilson, 1975), that genes and evolutionary forces influence behavior. This is necessarily true for any heritable behavior with implications for survival or reproductive success, even a given effect is small (Penke et al., 2007). This applies to animals that live in social groups with cooperation and competition as necessary ingredients for survival and success, such as human beings. Leadership, in particular, may be an important evolutionary context (Van Vugt, 2006; Van Vugt et al., 2008).

For instance, consider leadership as an example of social rank. Rank in social hierarchies is very important to social functioning in several primate species. For instance, young adult male chimpanzees spend tremendous amounts of time and effort in attempts to ascend the social ladder to attain alpha male status (Wrangham and Peterson, 1998) and low rank in the social hierarchy has severe negative implications for stress and health in savannah baboons (Sapolsky, 2001, 2005). Such studies provide a useful context for considering leadership behavior. Our distant primate cousins may shed light on aspects of social behavior, stripped of human cultural context, that are shrouded in complexity for humans. Such comparative approaches may aid us in understanding the origins and functions of leadership in our evolutionary past. Similar, though non-identical, evolutionary pressures are likely to have shaped such behaviors in the great apes.

Such observations about social rank in primate species become important when we consider the first finding of importance to the sociogenomic approach—that the genome is highly conserved across species. Because of this, we can learn a great deal about human behavior from animal models, a point we return to shortly. There has already been some effort along these lines in personality psychology (e.g., Gosling, 2001, 2008; King et al., 2005; Mehta and Gosling, 2006). We believe that a great deal can be learned regarding human leadership and influence processes by examining these processes in other species, and some compelling work has already been done (e.g., de Waal, 2000; Arvey et al., 2014). Furthermore, animal models can provide extraordinary isolation of variables. By studying leadership in chimpanzees we can see the political process stripped of the artifacts of human cultures and language.

Sociogenomics provides a deep reason for examining behavior comparatively: due to the conservation of the genome, behavior syndromes in multiple species probably share genetic determinants and molecular pathways (e.g., Donaldson and Young, 2008). Work that might not be possible with human subjects may be possible using animal models. That is, using current technology, barring post-mortem autopsies, it is not possible to examine gene expression levels in the human brain, but the relevant molecular pathways may be examined in surrogate animals, such as rats and mice.

**Figure 1:**
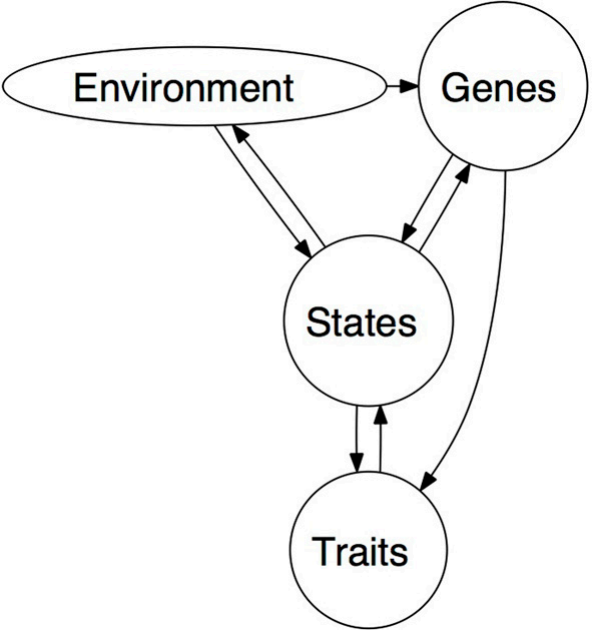
**Sociogenomic model of traits (adapted from Roberts and Jackson, 2008)**.

The second finding is even more relevant in comparison to other contemporary models of the genetic determinants of behavior. The effects of genes are dynamic in their transactions with the environment: genes in themselves *do not determine* behaviors, thoughts, or feelings. Genes code for proteins, period. They do not directly encode behavior; rather, genes are expressed via the proteins for which they code. The general process is as follows: genes are transcribed into RNA sequences that are then translated into polypeptides, and these finally form proteins. The amount, location, and timing of the production of proteins are contingent on the cellular environment. The cellular environment is influenced by the external environment at every step of the above process. The processes of gene expression link the influence of DNA with the environment (Robinson, 2004). This is in contrast to the “genes as distal causes” approach outlined in Figure 1. Unlike Nicholson's (1998) admonishments that leader characteristics are fundamentally innate, but can be moderated by the situation, a sociogenomicist realizes that genes may also moderate responses to the environment. That is to say, the environment may have a direct effect on behavior, and genes may modulate that environmental effect. Genes can be both causal drivers that the environment constrains, but it is also possible for the environment to be the causal driver that genes serve to constrain (cf., Robinson et al., 2008).

## Gene expression

We first note that gene expression is a complex phenomenon: we will often discuss “which genes are expressed,” but this is short hand for the degree to which genes are expressed. When it comes to behaviors such as leadership or job performance, the differences we discuss are more typically quantitative rather than qualitative. The location (in the brain) of the gene being expressed or the degree of expression are the key features^1^. There are two major mechanisms that account for differences in gene expression. The first mechanism is differences in genetics between people, which are consistent with the “Leaders are born” (nature) position. The second mechanism is that gene expression can be influenced by variations in environmental conditions, consistent with the position that “Leaders are made” (nurture). Both of these mechanisms may result in different levels of gene expression, meaning that both affect which proteins are being synthesized in the person at any given time, and most importantly meaning that both can affect the neurobiology associated with leadership behaviors and traits.

These two mechanisms are so tightly intertwined that it is absolutely untenable to frame the question whether leaders are born vs. made, or even to simply assert that they are born and made. A dichotomous viewpoint is demonstrably false. Both the genetic mechanism and the environmental mechanism operate on the same substrate: the genome itself. We cannot emphasize this point strongly enough. Environments wield their influence by affecting the production of proteins—gene expression. Both are capable of influencing gene expression and both can affect brain functioning similarly. We believe that nature and nurture should be viewed not as two distinct processes but merely two sides of a coin (Robinson, 2004; Balaban, 2006; Roberts and Jackson, 2008). For instance, consider the study of genetics and social environment by Zhang et al. (2009b). The genetic influence on leader role occupancy is strongest in low socioeconomic strata and weakest for those brought up in highly enriched environments. Genetic differences may predispose someone to be a good leader but a certain environment may squash this or a person born with unfavorable genetic polymorphisms may live in an enriched environment and become successful. The key issue in sociogenomics is how genes and environmental experiences combine together in their effects.

Think of social status as an environmental variable. Social status can have profound physiological effects. As an example, consider the orangutan. Dominant males have pronounced secondary sexual characteristics, but subordinate adult males have their development arrested in a “subadult” state (Maggioncalda et al., 2002). This is not just a chronological phase in their development; should the dominant male be removed from power, the subordinate males will develop secondary sexual characteristics. Note that the subordinate males are not truly juvenile; they are fertile and can reproduce, generally by forcing intercourse with females when the dominant male is absent (Sapolsky, 2005). In this case, an environmental variable, social status, greatly affects a physiological mechanism—physical maturity.

In a similar vein, Roberts and Jackson (2008) describe a particularly dramatic example of the impact of the environment on gene expression: the life course of the blue-headed wrasse, a tropical reef fish (e.g., Stearns, 1992). The males are large and bright blue, while the females are small and dull brown. Males tend to collect a harem of females whom they protect and mate with. When a predator eats the male, the females do not search out a new male. One of them instead transforms into a male overnight. This effect is genetically mediated, but it is accurate to say that an environmental condition, loss of the harem's male, causes the sex of the fish to change. That is, an environmental event induces a change in gene expression, which then results in profound physical and behavioral changes.

Further examples of genetic and environmental forces working together can be drawn from the lives of honeybees. Worker bees begin life as caretakers of the hive but eventually become food gatherers (Robinson, 2004). This change is associated with changes in the expression of more than 2000 genes (Whitfield et al., 2003). Changes in the *for* gene are associated with shifts in the environment. For instance, when there is a shortage of food gatherers, the *for* gene becomes expressed and a cascade of changes occur that transition the worker into a food gatherer (Ben-Sharar et al., 2002). The gene is similar across all bees, but the influence of the gene for a particular bee is contingent on the state of the particular bee's environment—the conditions of its hive.

Such changes in gene expression that are not dependent on the DNA sequence itself are called *epigenetic* effects (Zhang and Meaney, 2010). Such effects occur as a result of various mechanisms, but the most well understood is DNA methylation. Methylation stops the transcription of a gene, halting production of the protein that gene codes for. Strands of DNA can continue to be methylated across time, demonstrating how an environmental effect continues to effect expression even after the environment is removed.

Such epigenetic effects can manifest in very subtle ways. For instance, rat pups that have been licked more by their mothers handle stress better than pups that have not been licked, but that licking behavior is itself heritable. So, it is unclear whether the response to stress in rat pups is directly heritable or if it is environmentally mediated by this licking behavior (Weaver et al., 2004)—the genetic and environmental effects are observationally confounded. Examining the mechanisms of gene expression clarifies this problem, however: maternal licking behavior affects methylation that in turn affects expression of the glucocorticoid receptor gene. Rats with greater activity in the glucocorticoid receptors are better able to tolerate stress. This means that the observed individual differences in rat stress response were not directly attributable to genetics, but via the epigenetic modification of gene expression due to methylation (Weaver et al., 2004). The effect of the gene is contingent on environmental factors.

There is some early evidence for such epigenetic effects in humans. For instance, methylation patterns between identical twins are highly, but not perfectly correlated (Mill et al., 2005). Identical twins share 100% of their genes, but this finding indicates that some life event(s) altered gene expression in the twins studied. This finding has been replicated, and it has been shown that the degree of epigenetic dissimilarity was correlated with the age of the twins and the amount of time the twins had spent together (Fraga et al., 2005). Older twins and twins who spent more time apart had greater differences in methylation patterns. Even for identical twins, who share exactly the same genetic material, external events are capable of changing the way these genes are expressed. DNA is not the only causal driver of gene expression; the environment can play an important role.

A sociogenomic leadership theory that embraces gene-environment interplay points to new avenues of research. For instance, consider the Avolio et al. (2009) study of the effects of authoritative parenting and rule-breaking behavior on leader role occupancy. A sociogenomic leadership researcher would be interested in the mechanisms by which authoritative parenting operates on rule-breaking behavior and leader emergence. Like the rat pups above, are certain gene sequences silenced by authoritative parenting? What mechanisms might drive these findings? Parenting style may set limits on the environments that a child is able to enter. This would be a case of the effect being entirely environmentally mediated, whereas the example of the rat pups is genetically mediated, but either mechanism is possible.

While it is not yet possible to study gene expression directly in living humans, studies of gene-environment interactions suggest that these contingencies may exist. Most behavioral genetic studies in psychology find that somewhere around half of the variance in phenotypes is genetic and the other half is mostly attributable to unique environmental effects (there is often some small amount of variance attributable to common environment found, but see Turkheimer's second law above). Such findings are often built around an improperly specified model: one that does not explicitly account for the environment (e.g., Brofenbrenner and Ceci, 1994). When the environment is explicitly taken in to account, the findings can be markedly different. The heritability estimates are moderated by the environmental effects, such that heritability can be higher or lower as a function of some environmental variable, such as the effects of socioeconomic status on the heritability of intelligence discussed above (Turkheimer et al., 2003). For instance, the heritability of negative emotionality decreases and the effect of *shared* environment increases at higher levels of parental conflict (Krueger et al., 2008).

Such findings from the personality psychology literature may help to put results such as Zhang et al. (2009a,b) into context. Recall that the genetic effect on leadership role occupancy was moderated by level of social enrichment. The sociogenomic approach leads to questions about how these effects occur. What mechanisms get under the skin, transmitting environmental effects to the genome? Roberts and Jackson (2008) presented a schematic model for a sociogenomic personality psychology. Figure 1 presents a modified version of this model. We consider all major facets of the model to be latent variables; we assume that even biological substrates will be measured with some error. What is important to note in this model is the direction of the arrows. The environment may act directly upon the biological substrates, through epigenetic mechanisms and gross insults (toxins, brain parasites, iron damping rods through the face), but the biological substrates act on the environment indirectly by way of traits and, most proximally, states. Environments may also act indirectly on the biological substrates via experienced psychological states. For example, the structure of the brain is reconfigured under long-term stress; the medial prefrontal cortex and hippocampus atrophy and the orbitofrontal cortex and basolateral amygdala expand (McEwen et al., 2006).

We argue that leadership style is essentially a trait, a pattern of behaviors that is relatively stable across time and situations. Individual leadership behavioral episodes, such as influencing a particular follower are states (cf., Fleeson, 2001; Beal et al., 2005; Fleeson and Leicht, 2006). Traits and the environment both affect states, and states act on the physiological substrates, which in turn influence trait levels. For example, individuals told to pose in powerful ways have been shown to experience elevated levels of testosterone and decreases in cortisol levels which, in turn, impacts their decision-making and risk-tolerance (Carney et al., 2010).

The key behavioral component of this model is the state: individual behavioral episodes. The individual engages in behaviors that set goals, build relationships, express trust in subordinates, initiate structure, and so on. These behavioral episodes are determined by environmental constraints (e.g., department policy, requests from senior management, compensation structure) and by traits (e.g., need for power, need for affiliation, dominance, sociability, attachment style, propensity to trust). From the standpoint of developing leaders, these episodes are key. Like stress remodeling the brain as described above, how can leader development interventions be constructed to redesign the neural architecture of the leader? We see the point of leader development interventions as using the environment to induce states that ultimately alter trait levels.

We believe that the key difference between the current models of biology employed by leadership researchers and the sociogenomic perspective is one of generativity. The sociogenomic perspective, as outlined above and summarized in Figure 1 provides direction to research investigating the genetic and environmental effects in leadership. We outline a few key areas for emergent scholarship below. Work under current models is effectively descriptive, documenting genetic and environmental effects. The unifying functional framework provided by sociogenomics presents many opportunities for exploration.

## Reconsidering the rectangle: born and made

The current approaches to the biology underlying psychological characteristics seem to view genetics as an unchanging causal force on behavior. In such conceptualizations, consequential social phenomena, such as leader effectiveness, lie at the end of a causal chain begun with the biological substrates underlying personality traits (e.g., McCrae and Costa, 1996; cf. McCrae, 2010). While other stages in the causal chain are seen as subject to environmental pressures, these biological substrates are not. DNA is the core of these structures, and is seen as an immutable influence on phenotypic traits throughout the lifespan. The assumption is that, as genetic polymorphisms do not change, the effects of DNA on behavior should be constant, therefore any changes in phenotype are caused by genes (McCrae, 2010).

**Figure 2:**
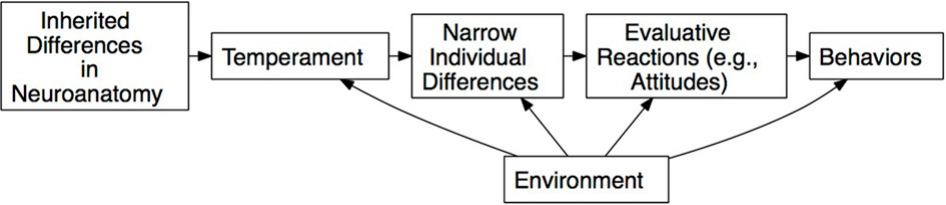
**Traditional biological model as discussed in Ilies et al. (2006)**.

Ilies et al. (2006) employed similar reasoning in their argument that causality flows from genetic factors through large, heterogeneous traits to narrower traits to behavior. Figure 2 presents a “born not made” model of leadership, adapted from Roberts and Jackson (2008). This model remains current in biological thinking throughout the social and organizational sciences. The origins of this perspective lie in Eysenck's (1967) views on personality and intelligence, which have been very influential on biological thinking in psychology. The details of specific biological models vary, but the take-home point regarding models of this form is this: causal flow is always from the biological substrate to the behavioral or social outcomes (e.g., McCrae and Costa, 1995, 1996; DeYoung, 2010). This point of view seems well represented in organizational research, with a model like this implicit in Ilies et al.'s (2006) review, and the explicit argument in Antonakis et al. (2010) that personality traits can be used as instrumental variables in many settings in organizational research, because their levels are set exogenously by genes. According to this theoretical point-of-view, the environment is generally viewed as capable of modulating anything causally downstream from the functional neuroanatomy, but does not generally impact genetic or physiological systems, barring gross injury (such as the well-known fable of Phineas Gage).

Nicholson (1998) provides a fairly clear summary of this viewpoint. He describes three hypothetical children from a hypothetical family, each with a radically different temperament: the first is introverted and grows up to be a research scientist, the second is talkative and grows up to be a salesperson, the last is even-tempered and grows up to be a schoolteacher. Nicholson states, “Evolutionary psychology tells us that each one of these individuals was living out his biogenetic destiny.” Personality dispositions are described as hardwired. Leadership skills can be taught, but the “passion to lead” is an innate difference (cf. Doh, 2003). Nicholson points out that possessing this genetic endowment may not always lead to successful leadership, though, as situational characteristics may necessitate some other set of traits. From this perspective, the biological component of behavior—the disposition—simply *is*, and is effectively immutable; situational characteristics merely determine whether expressing that disposition is effective vs. ineffective. That is, genes are the causal drivers and the environment acts only in modifying the effectiveness of genetically caused preferences.

How should the behavioral genetic evidence discussed above be interpreted under this perspective? The key to understanding this perspective is that physiological systems are given causal primacy over psychological mechanisms. This is the approach adopted in some quarters of personality psychology, with the implication that genetic polymorphisms manifest themselves in specific neuroanatomical structures which in turn give rise to largely static psychological characteristics (McCrae and Costa, 1995, 1996; DeYoung, 2010). Advocates of this viewpoint usually argue that since the genetic polymorphisms are invariant and, barring gross injury, so are the neuroanatomical structures and their concomitant temperaments/traits. The environment is essentially restricted to affecting what McCrae and Costa refer to as *characteristic adaptations*, the learned habits that individuals develop to express their native traits in acceptable or functional ways within their environment.

The “Leaders are BORN” view tends to force such dichotomous thinking, however (and so does the “Leaders are MADE” perspective). If the genetic polymorphisms one is born with are invariant over the life course and they guide the development of the neural architecture we think with, how can it be otherwise? Researchers operating within this framework have a tendency to demonstrate that a genetic component exists for leadership (e.g., Johnson et al., 1998) or to statistically control for genetics in order to more purely estimate the environmental effects of interest (Avolio et al., 2009). The next section argues that this point avoids addressing the true complexity of the relationship between genetics and experiences as causal agents. Studies such Avolio et al. (2009) demonstrate a conceptual weakness in other interactionist perspectives, relative to the sociogenomic outlook. Statistically controlling for a genetic effect while estimating the environmental effect separates two inseparable things—remember both genes and environment operate through mechanisms of gene expression—and assumes that the genetic effect is invariant over time. These traditional interactionist studies ask the aforementioned question, “which contributes more to the area of a rectangle, its length or its width?” Like Nicholson (2005), we believe that a *truly interactionist* perspective is needed, but we believe that a sociogenomic approach—where both genes and environment are truly causal mechanisms—provides that perspective. Specifically, recent evidence indicates the epigenetic mechanisms are active throughout the life-course (Zhang and Meaney, 2010; Charney, 2012). It is possible that these mechanisms are responsible for various aspects of development and behavioral plasticity.

## What can organization studies scholars learn from the sociogenomic perspective?

The major take home messages from the sociogenomic perspective are quite broad. Sociogenomics provides a meta-theoretical framework that can assist leadership scholars in framing and interpreting new and existing research. This framing is achieved by recognizing the deep interdependence of genes and the environment in facilitating behavior. To explore this interdependence, we propose three platforms of research informed by the sociogenomic perspective.

### Proposal 1: conduct cross-species studies of social influence processes

Recall that one of the foundational points of the sociogenomic perspective is the conservation of the genome. One main point is that the behavioral syndrome we call leadership has direct analogs in other species, and that the chemical pathways that lead to the syndrome are likely to the same, whether the subjects of study are people, primates, or stickleback fish. Additionally, even simple species, such as nematodes, fruit flies, and honeybees, display interesting social behavior that have human analogs (Sokolowski, 2010). Roberts and Jackson (2008) pointed out that a sociogenomic personality psychology would be a comparative psychology from the start. This point is also true of a sociogenomic leadership theory. This can be a challenge because of the definition of leadership in many areas of biology—for instance, in behavioral ecology, leadership is often being the individual who selects which direction the group will move in most frequently (cf., Van Vugt, 2006), though this may provide some insight into humanity's evolutionary past.

To use this proposition, organizational researchers who embrace the sociogenomic model would first investigate how the behavioral syndromes associated with leadership roles are manifested in other species. For instance, the political rivalries and power plays within a colony of chimpanzees may inform research on power and status motives in human leaders or the process of coalition building in human work teams (de Waal, 2000). With this suggestion, we do not just mean to address neurobiological systems. Animal models may allow us to formulate tighter hypotheses about important experiences and environments. By examining the more visible social and power relations in animal models, we may have a better idea of whether important experiences or developmental environments occur early or later in life, whether those experiences involve peers, and the degree to which a formative experience can shape an individual. Social experiments that may be difficult or unethical with human participants might be possible. For instance, what happens both socially and neurobiologically when an individual at the top of a hierarchy in a particular context is moved into a new context? Are they still “a leader”; how is their neurobiology affected?

Such a lack of normal social context for leadership using animal models may disconcert many organizational scientists. We do not suggest that *normative* findings will be discovered from cross-species research; to expect so would mean we have committed the naturalistic fallacy—“that which is, must be good.” We suggest, instead, that such research can open up a very clear view of the neurochemical mechanisms that drive certain aspects of leadership-relevant behavioral syndromes. This, in turn, may provide deeper insights into the psychological mechanisms that constitute leadership. Additionally, the insights gained from understanding animal nature may have direct practical implications, for the opposite reason of the naturalistic fallacy. These insights may help us to understand how humans *want* to behave, contrary to organizational and societal expectations (e.g., de Waal, 2000; Van Vugt and Ahuja, 2011).

### Proposal 2: increase precision and specificity in measuring organizational behavior constructs

Part of the problem in asking strong biologistic questions about leadership is that leadership, as a set of phenomena, is likely too complex to submit to localization in specific neural structures or processes. A sociogenomic approach to leadership would thrive on detailed, specific measurements of its constructs and the differences between them. There are two major reasons for this. The first is that it is necessary to clearly understand the phenotype in order to progress in understanding the genotype—and its transactions with the environment. Measurement is, unfortunately, not a particular strength of current leadership research, and is weak in much of organizational behavior. The proliferation of constructs in leadership theory, with little clear evidence for their distinctiveness makes this point problematic: what are the important, distinct behavioral syndromes that sociogenomic researchers should be investigating? For instance, it has been shown that satisfaction with one's job has a heritable component (Arvey et al., 1989). That such attitudes are heritable has sometimes been explained by the heritability of more general personality traits (e.g., Olson et al., 2001), but is that argument fully consistent with a sociogenomic analysis (cf., Roberts and Jackson, 2008)?

In leadership research, specifically, another rationale for improved psychometrics in organizational behavior is that precise measurement would allow the community of leadership researchers to build up a well-specified nomological network to enable understanding of how, why, and when good leaders emerge and how they behave while holding their leadership roles. That is to say, what are the biological and psychological factors that predict “leadership”—before the putative leader is even thrust into any leadership role? Measurement in the field of leadership must be put on firmer psychometric grounding. Leadership scholars may need to invite assistance from psychometricians to achieve this goal. Even with such assistance, confronting biological systems may require further refinement of measures.

For example, serotonin functioning is implicated in dominance behavior in chimpanzees, and treatments with the serotonin precursor tryptophan increase dominance in everyday social interactions in humans (Moskowitz et al., 2001). In particular, to understand the role played by serotonin, one needs to differentiate between two modes of self-regulation. In the first mode, individuals engage in quick, affect-laden responses built upon approach and avoidance emotions (e.g., joy vs. fear). The second mode is an effortful control system that can serve to guide voluntary behavior or to inhibit inappropriate responses. The second mode is capable of overriding the first mode. Essentially, at any given time, any given human is working in one of two ways: a highly emotional, reactive mode or a deliberative, thoughtful mode. Carver et al. (2008) argued that the serotonin system facilitates greater effortful control.

Using such a highly specific, detailed formulation of the constructs allows considerable insight into the serotonergic system and its associated behavior syndromes. Depression reflects the combination of low activation in both the approach system and the effortful control system. Similarly, the construct *impulsivity* confounds high activation in the approach system with low effortful control. Since serotonin facilitates effortful control, it therefore affects a wide range of seemingly unrelated psychological domains, such as depression, angry hostility, and impulsivity.

Current assessment of leadership is frankly weak from a biologically informed perspective. Behavioral syndromes such as transformational, transactional, or authentic leadership are probably too coarse (see Avolio and Gardner, 2005) to be diagnostic of the physiological systems at play. We do not mean to single out these constructs as being the only ones in the leadership literature that are too broad to aid in building biological theories of leadership. It is unlikely many of the leadership measures in current widespread use would be sufficiently precise for such purposes.

To reiterate, there are multiple ways to think about leadership. In this essay, we have approached leadership in a behavioral or trait-like manner. That is, that a leadership style is a pattern of behaviors exhibited by an individual in a formal or informal leadership role that is fairly stable (in contrast to, say, emotions) across time and situations^2^. We wish to be clear that we are not indicating that leadership *per se* is a trait, but that a variety of leadership constructs, such as leadership style, can be approached in the same manner as other individual differences.

Beyond the previous considerations, the individual's physiology has implications for any conceptualization of leadership, and the measurement systems used should incorporate those considerations. As an example, consider the serotonergic system. It is implicated in dominance, and dominance appears important to attain and maintain status. Now, we are left with a host of research questions regarding the role the serotonin system plays in dominance and status attainment. For example, how does variability in serotonergic functioning affect leader emergence? Does attaining leadership status, in turn, affect the serotonergic system (a corresponsive effect; Roberts and Caspi, 2003)? Different social settings are likely to allow only some dominance displays—what role does serotonin play in navigating this social milieu?

A connected point that is important here is that multiple methods should be used to investigate the biological underpinnings of leadership behavior. Hormonal assays can be used to study the roles that stress and sex hormones play in various leadership-relevant interpersonal interactions. For instance, recent work shows that while member testosterone, as measured with saliva swabs, does not predict member status within the group, mismatches between testosterone levels and member status in group settings negatively impact the group's collective efficacy (Zyphur et al., 2009). There are also indirect measures of testosterone level that can predict leadership-relevant qualities. Facial masculinity, a signal of testosterone, is associated with rank both at US Military Academy at West Point and late-career rank (Mueller and Mazur, 1996). Depth of voice, another indicator of testosterone, is a robust signal of dominance (Wolff and Puts, 2010).

We also think that brain-imaging work can be helpful in clarifying the meaning of leadership constructs. Use of brain imaging methods is tightly tied to our concerns regarding the specificity of measurement systems employed in leadership research. For instance, is it meaningful to ask, what are the neural correlates of transformational leadership? As an example, it has been suggested that neuronal coherence (an index of communication between areas in the brain) in the right frontal cortex may be associated with visionary communication (Waldman et al., 2011). Perhaps more meaningful is to narrow this question down to deal with the construct of “charisma” (Gardner and Avolio, 1998). The point remains that constructs must be sufficiently well defined so as to permit investigation of their neural substrates. Additionally, we can ask this question in two ways. First, on the leader side, which neural mechanisms are involved in the kinds of idealized influence tactics that constitute charismatic leadership? Secondly, on the follower side, which mechanisms do those influence tactics engage?

Finally, finer measurement of leadership constructs would increase the utility of molecular genetic studies. For instance, consider the measurement of power motivation, which has been argued is extremely important to the acquisition of leader status (Nicholson, 1998; Pfeffer, 2010). Power motivation can be measured using an approach motivation framework, desire for power, or using an avoidance motivation framing, fear of power (Harms and Roberts, 2006; Harms et al., 2007). Using these approaches may help to clarify the role of the neurophysiological systems in understanding leadership phenomena, and help to direct attention to candidate genes (such as dopamine receptor and serotonin transporter genes). The molecular genetic approach is open to criticism, in that the results are notoriously difficult to replicate—but the original research should be done so that issues of replication can even be addressed.

### Proposal 3: identify key contexts and timing for adult development at work

The key insights from a cross-species, sociogenomic view of leadership demonstrate how critical particular environmental experiences can be for profound behavioral—and sometimes physical—change. Avolio (2005) discussed the tension between the “born” and “made” perspectives in the development of leaders. The premise is that the genetic endowment an individual has is a starting point. The stream of events and situations a person experiences is what develops the individual as a leader. The key insight from sociogenomics is that, even for highly heritable traits, those traits are still open to environmental influence. Again, heritability does not reflect the degree to which an attribute is “set in stone” and does not necessarily act as a constraint on the amount of influence that environment *can* have on shaping leadership. That is, no matter how high the heritability there is still a possibility for environmental interventions. Thus the question becomes, what are the situations (occurrences, times of life, and so on) that will allow a person to develop into a leader and are there interventions that can lead to better leadership?

A sociogenomic leadership approach would help to develop a science of leader development in two key ways: to help understand what contexts are developmentally important and when they can be expected to occur (cf., Day et al., 2009). For instance, it is appropriate to question what the evolutionarily appropriate contexts for leader development are. Hogan (2007) has argued that in the work context, individuals must balance two fundamental motives: getting ahead and getting along. Hogan argues that these motives are products of our evolutionary history as social animals. Entry into the organization can be viewed as entry into a social hierarchy, and many of the situations that follow can be viewed through the lens of attempts to attain and maintain status within the hierarchy. Again, comparative study of other primates or traditional social groups (e.g., hunter-gatherers) could help us understand these contexts.

What if it is possible to design interventions that counteract or decrease the phenotypic variance attributable to genes (similar to the Turkheimer SES and IQ studies mentioned above)? For example, consider the US Military Academy at West Point. West Point has a strict organizational hierarchy, with cadets attaining various ranks that mirror the active duty Army. Additionally, West Point has the explicit goal of *developing* cadets into military leaders, and uses a variety of formal and informal developmental interventions to do so, including 360 feedback mechanisms. There are individual differences in the developmental trajectories for cadets for scores on those 360 instruments (Harms et al., 2011), indicating that some cadets are more successful at navigating this formal hierarchy. A sociogenomic approach to such a study would attempt to capture the psychological, physical, and political tools that cadets use to navigate the organizational hierarchy, and how those tools relate to leader competencies across time. For instance, do leadership skills enable assent in the organization, or does role attainment facilitate skill development? Another key question is how do experiences in the organization get translated into trait-like leadership competencies; what behavioral episodes are key?

As a further example, can traumatic experiences catalyze the development of leadership within a person, such that a person experiencing traumatic events becomes more resilient and more capable of exerting leadership (e.g., Avolio, 2005)? A sociogenomic researcher might ask which genes are expressed (or suppressed) when trauma occurs? What is the biochemical pathway such trauma induces—does the expression of these genes trigger a cascade of expressions in other genes that influence activity in multiple areas of the brain? For instance, trauma is implicated in a number of negative behavioral syndromes, such as antisocial personality disorder and depression (Caspi et al., 2002, 2003). What are the physiological differences that allow some individuals to use traumatic events to catalyze their leader development, as opposed to sinking into violence or despair? What interventions can alter the individual's reaction to the traumatic event? Can we identify the molecular pathways such an intervention would engage? How does the whole process play out? Understanding the biological mechanisms that mediate the effects of trauma and recovery will help design more effective interventions. Based on the model in Figure 1, it is clear that because psychological states mediate the influence of the environment on both the biological substrate and leadership-relevant traits, it is likely that effective leadership interventions should be sustained over longer periods of time. For instance, the West Point study by Harms et al. (2011) found development on leadership competencies persisted over a period of 2 years.

Furthermore, the existing evidence from behavioral genetic studies shows that a considerable amount of variance in leadership outcomes is unexplained. Unique environmental factors explain most of the variance in leader role occupancy, but only a fraction of this variance has been explained by measured life experiences (Arvey et al., 2007). How might experiences with authority, early leadership roles, responsibility in fraternal, social, or civic organizations, and other life experiences shape the states that individuals experience? How do those states affect gene expression and neural architecture? Is it possible to use animal or ethological models to identify important roles and timing for leader development experiences? We focus above on leader development, but it seems clear that the roles, demands, and general characteristics of an individual's job impacts his or her personality development (e.g., Roberts, 2006). If personality is important to a wide variety of on-the-job behaviors, then this development will have important consequences of our understanding of the relationship between genetic, neurological, and behavioral variables in organizational settings.

### Proposal 4: closer integration with evolutionary psychology

Up until this point, we have largely ignored the other main biological research tradition in behavioral science: evolutionary psychology. One reason is that, until relatively recently evolutionary psychology has focused on species-general adaptations (i.e., mechanisms or structures that do not vary over individuals in a population; e.g., Tooby and Cosmides, 1990; cf., Penke, 2010), and such universal features are less generally relevant in organizational contexts: understanding them could help design very general aspects of the work environment (e.g., safety, compensation systems), but are less helpful in selecting, training, motivating, or leading individuals at work. More recently, though, researchers have begun to integrate evolutionary psychology with research on individual differences (e.g., Penke, 2010; Buss and Penke, 2014). Such efforts revolve around understanding individual genetic variation and its impacts on behavioral characteristics. We have argued throughout this essay that sociogenomics is an effective meta-theoretical framework for studying psychological, behavioral, and neuroscientific phenomena in organizations; evolution (and, by extension, evolutionary psychology) is *the* meta-theory that the sociogenomic framework plugs into (cf., Buss, 1995).

Evolutionary theory also provides a variety of conceptual tools that can aid researchers in analyzing problems and behaviors, such as life history theory and costly-signaling theory, to name just two (Buss and Penke, 2014). Consider life history theory, as an example. Individuals have limited time and energy to devote to their various pursuits, and so face trade-offs when investing these resources in any particular activity. Life history theory provides a broad framework for analyzing these choices (Kaplan and Gangestad, 2005). For example, an individual male may invest efforts into securing a leadership position at work to increase his status and compensation, in order to secure a desirable mate and provide resources for future offspring, helping to solve the two major problems of *reproduction* and *parenting* (cf., Buss and Penke, 2014). Thinking about the action *acquiring a leadership position* in this way could help to better understand the motivations and cognitive processes the individual has, opening this action up to greater theoretical elaboration.

Further, evolutionary theory can help to provide implementation guidelines for our previous proposals. Specifically, consider our discussion of *identifying key contexts and timing for leader development* above. Such contexts are situations, in the classical person-situation debate sense (cf., Mischel, 1968). Important situations are defined by the adaptive problems that obtain within their boundaries (Buss and Penke, 2014). A relevant context for leadership development may be a child's first day of school, for instance: his or her first exposure to a prominent status hierarchy with authority figures (i.e., teachers, administrators) who are not the child's parents. While we focus here on the first day of school, it is the experience of the status hierarchy that defines the *evolutionarily* important context.

## Ethical considerations

In some ways, genetic or other physiological screening in organizations is similar to the psychological and physical testing already used for selection among applicants (cf., Guion, 1998). The measures used in those settings, such as cognitive ability tests, personality assessments, and tests of physical strength, dexterity or endurance are imperfect indicators of the underlying psychological or physical entity (cf., Lord and Novick, 1968). They are also imperfect predictors of future behavior at work. Often, however, the results of these measures are imbued with a certain physical, biological interpretation: that is, a person's levels of some personality trait, like conscientiousness—the tendency to be neat, orderly, punctual, achievement-oriented (cf., Barrick and Mount, 1991)—is set by the person's genes (e.g., Antonakis et al., 2010). If that viewpoint held, then direct assessments of the genes or neuroanatomical structure that serve as the biological foundation of conscientiousness would be just as appropriate.

One of the major purposes of this review has been to demonstrate *why* that view has flaws. Possessing a particular genetic polymorphism seems unlikely to be enough to determine an individual's standing on a trait as complex as conscientiousness (or any other complex behavior). The genes a person possesses may express themselves differently (or not at all) conditional on the environment. Further, environmental changes may impact the individual's psychological states, which could then affect gene expression and remodel the person's neuroanatomy (cf., Roberts, 2006; Roberts and Jackson, 2008). When these possibilities are taken into account, it seems unwise to simply examine an individual's current biology and make strong behavioral predictions based on it.

Let us return to the example from the beginning, of the “psychopath” neuroscientist, James Fallon. If we lived in a world with rigid genetic or neuropsychological screening, he would perhaps never have been admitted to graduate school to earn a Ph.D. We would then not have his example to illuminate the possibility that our genes are not our destiny, that an individual whose genes appear to code for psychopathy, and whose neurological functioning bears this out, can be a successful scientist with a close family. Under the sociogenomic framework, there is a complex path from the particular variant of a gene that an individual possesses and the behaviors they are likely to exhibit; as a result, it seems to us that organizational interventions based on genetic or neurological information are a long way from being tools in the practicing manager's kit.

## Conclusion

This paper is meant to incorporate theoretical insights from molecular biology within leadership research, using a framework that has been profitable to understand social behavior across species, time and outcomes (Robinson, 2004; Robinson et al., 2008; Bell and Robinson, 2011). Certainly, we do not cover every aspect of this theory, nor can this be considered the final word on the topic. We mean to contrast static thinking regarding the influence of both traits and genetics with the highly transactional view of the gene-environment interplay provided by the sociogenomic perspective. That is, to say that a characteristic is genetic is not to say that it is unchanging; there is a fundamental interplay between genes and the environment throughout the life course. Our genetic material does not make our destiny; it does not have a simple direct influence on phenotypic behavior. Sociogenomics encourages leadership researchers to focus on functional questions: what mechanisms facilitate leader emergence? What psychological adaptations facilitate effective leadership? What are the physiological substrates of leadership constructs?

Further, sociogenomics urges leadership researchers to attend to the evolutionary context in which leadership emerged: this may provide key insights into how these functional mechanisms operate within modern organizational contexts. For instance, how is social status attained within an organization, and which mechanisms facilitate its attainment? A sociogenomic leadership theory would provide a modern biological framework for interpreting genetic research in leadership by encouraging detailed research questions regarding the mechanisms underlying genetic and environmental effects found in contemporary behavioral genetic studies.

Recent interest in and efforts to incorporate biological reasoning into management and leadership seem to point to a bright future. To this end, we have borrowed and elaborated on a theoretical model from biology. This is a model that has some traction in disciplines that have close ties to leadership theory, most notably personality psychology. We advocate a move to a sociogenomic leadership theory. The perspective offered by this model shows us that DNA is not always the causal driver of behavior. Environmental conditions interact with genes to build the biological architecture upon which behavior plays itself out.

### Conflict of interest statement

The authors declare that the research was conducted in the absence of any commercial or financial relationships that could be construed as a potential conflict of interest.

## Footnotes

^1^We thank an anonymous reviewer for clarifying this point to us.

^2^Alternative views of leadership may conceive of leadership as a social process centered around influence or as a relationship. However, we believe that the most popular operationalizations of leadership (i.e., self- and other-reports of typical behaviors) are consistent with a trait-like perspective of leadership, where traits are considered as typical levels of behavior that persist over time and situations, but are flexible and develop over time, rather than being static (Roberts, 2006).
